# Linalool, a Fragrance Compound in Plants, Protects Dopaminergic Neurons and Improves Motor Function and Skeletal Muscle Strength in Experimental Models of Parkinson’s Disease

**DOI:** 10.3390/ijms25052514

**Published:** 2024-02-21

**Authors:** Wan-Hsuan Chang, Hung-Te Hsu, Chih-Cheng Lin, Li-Mei An, Chien-Hsing Lee, Horng-Huey Ko, Chih-Lung Lin, Yi-Ching Lo

**Affiliations:** 1 Department of Pharmacology, School of Medicine, College of Medicine, Kaohsiung Medical University, Kaohsiung 80708, Taiwan; u109800003@gap.kmu.edu.tw (W.-H.C.); anlm@kmu.edu.tw (L.-M.A.); 2 Graduate Institute of Medicine, College of Medicine, Kaohsiung Medical University, Kaohsiung 80708, Taiwan; cllin@kmu.edu.tw; 3 Department of Anesthesia, Kaohsiung Medical University Chung-Ho Memorial Hospital, Kaohsiung 80756, Taiwan; hdhsu@kmu.edu.tw; 4 Faculty of Anesthesiology, School of Medicine, College of Medicine, Kaohsiung Medical University, Kaohsiung 80708, Taiwan; 5 Department of Pharmacology, School of Post-Baccalaureate Medicine, College of Medicine, Kaohsiung Medical University, Kaohsiung 80708, Taiwan; chlee0818@kmu.edu.tw; 6 Department of Medical Research, Kaohsiung Medical University Hospital, Kaohsiung 80708, Taiwan; 7 School of Pharmacy, College of Pharmacy, Kaohsiung Medical University, Kaohsiung 80708, Taiwan; hhko@kmu.edu.tw; 8 Division of Neurosurgery, Department of Surgery, Kaohsiung Medical University Hospital, Kaohsiung 80756, Taiwan; 9 Department of Surgery, School of Medicine, College of Medicine, Kaohsiung Medical University, Kaohsiung 80708, Taiwan; 10 School of Post-Baccalaureate Medicine, College of Medicine, National Sun Yat-Sen University, Kaohsiung 80424, Taiwan

**Keywords:** linalool, Parkinson’s disease, neuroprotection, antioxidant, motor activity

## Abstract

Parkinson’s disease (PD) is a common neurodegenerative disorder characterized by the gradual loss of dopaminergic neurons in the substantia nigra pars compacta (SNpc), resulting in reduced dopamine levels in the striatum and eventual onset of motor symptoms. Linalool (3,7-dimethyl-1,6-octadien-3-ol) is a monoterpene in aromatic plants exhibiting antioxidant, antidepressant, and anti-anxiety properties. The objective of this study is to evaluate the neuroprotective impacts of linalool on dopaminergic SH-SY5Y cells, primary mesencephalic and cortical neurons treated with 1-methyl-4-phenylpyridinium ion (MPP^+^), as well as in PD-like mice induced by 1-methyl-4-phenyl-1,2,3,6-tetrahydropyridine (MPTP). Cell viability, α-tubulin staining, western blotting, immunohistochemistry and behavioral experiments were performed. In MPP^+^-treated SH-SY5Y cells, linalool increased cell viability, reduced neurite retraction, enhanced antioxidant defense by downregulation of apoptosis signaling (B-cell lymphoma 2 (Bcl-2), cleaved caspase-3 and poly ADP-ribose polymerase (PARP)) and phagocyte NADPH oxidase (gp91^phox^), as well as upregulation of neurotrophic signaling (brain-derived neurotrophic factor (BDNF) and nerve growth factor (NGF)) and nuclear factor-erythroid 2 related factor 2 (Nrf2)/heme oxygenase-1 (HO-1) pathway. In MPP^+^-treated primary mesencephalic neurons, linalool enhanced the expressions of tyrosine hydroxylase (TH), Sirtuin 1 (SirT1), and parkin. In MPP^+^-treated primary cortical neurons, linalool upregulated protein expression of SirT1, γ-Aminobutyric acid type A-α1 (GABA_A_-α1), and γ-Aminobutyric acid type B (GABA_B_). In PD-like mice, linalool attenuated the loss of dopamine neurons in SNpc. Linalool improved the motor and nonmotor behavioral deficits and muscle strength of PD-like mice. These findings suggest that linalool potentially protects dopaminergic neurons and improves the impairment symptoms of PD.

## 1. Introduction

Parkinson’s disease (PD) is a complex, multifactorial disease characterized by regression of dopamine neurons in the substantia nigra pars compacta (SNpc) [1]. Accumulating evidence suggests that several factors, such as neuroinflammation, mitochondrial dysfunction, oxidative stress, and apoptosis of dopamine neurons, play roles in the progression of PD [2,3]. The neuropathological changes eventually lead to clinical symptoms in PD patients, including rigidity, resting tremor, postural instability, and slow movement [4,5]. In addition to these typical characteristics, PD patients also manifest nonmotor symptoms [6], including autonomic dysfunction, anxiety, depression, and sleep disturbance [7], contributing to a decline in the quality of life for individuals with PD.

Apoptosis is a regulated cell death process that cells undergo to adapt to the dynamic living environment by eliminating useless or harmful cells. Essentially, apoptosis is a beneficial outcome of gene regulation [8]. However, in diseased conditions, apoptosis can become dysregulated, leading to the damage of normal cells and exacerbating the progression of the disease. Research has indicated a significant presence of apoptotic neurons in the midbrain of individuals with PD. The progression of PD involves causative factors triggering the apoptosis of otherwise healthy neurons [9]. Consequently, apoptotic signaling exacerbates the risk of additional pathological events, including neuro-inflammation, contributing to further damage to neurons. Neurotrophic factors have been established to play an important role in the survival of cells in PD [10]. These factors, like brain-derived neurotrophic factor (BDNF) and nerve growth factor (NGF), were dramatically diminished in the nigra of the human brain inflicted with PD [11]. Meanwhile, the reduction of plasma levels of BDNF and NGF has been shown in bipolar disorder and manic and depressed patients [12,13,14]. NGF was shown in some studies to be able to protect or restore dopamine neurons after injury. BDNF is more clearly associated with the nigrostriatal dopamine system. BDNF increases the survival of cultured dopaminergic neurons and stimulates neurite outgrowth and arborization. Therefore, prevention of BDNF downregulation is neuroprotective for dopamine neurons [15].

The brain, an organ with a high oxygen consumption rate, is considered highly susceptible to damage from oxidative stress, which has been proposed as an essential factor in the progression of PD [16]. Oxidative stress results from an imbalance between antioxidant defense systems and the generation of reactive oxygen species, leading to neuronal death or neurodegeneration [17]. Nuclear factor erythroid 2–related factor 2 (Nrf2) is a transcription factor sensitive to redox reactions, inducing the expression of a series of antioxidant proteins to reduce cell damage caused by oxidative stress. Moreover, overexpression of Nrf2 downstream gene Hemoxygenase 1 (HO-1) has produced a proper survival response against oxidative stress in PD [18,19,20]. Therefore, antioxidant therapy targeting Nrf-2 and HO-1 might be essential in preventing and treating PD.

**Figure 1:**
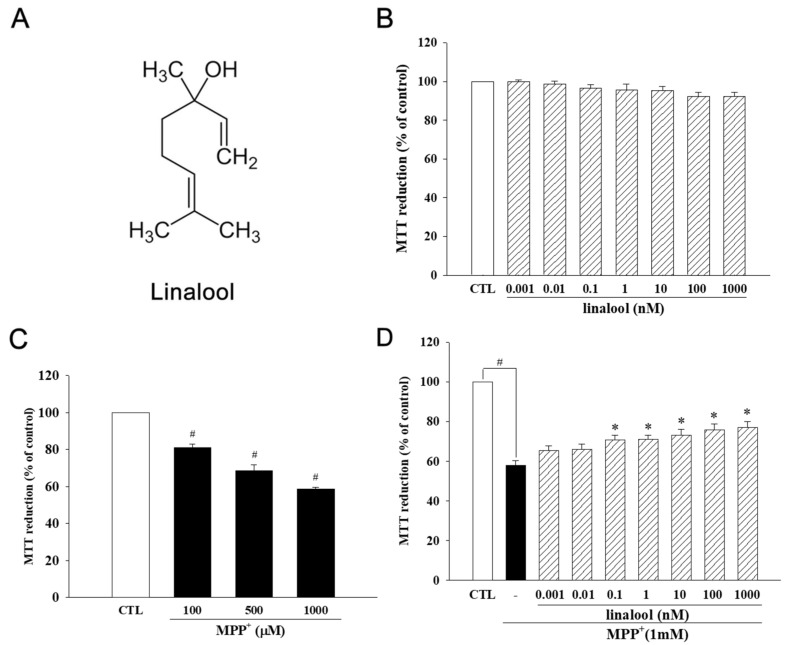
Linalool attenuated 1-methyl-4-phenylpyridinium (MPP^+^)-induced cytotoxicity in SH-SY5Y cells. (**A**) Chemical structure of linalool. (**B**) Effects of linalool on cell viability of SH-SY5Y cells. SH-SY5Y cells were treated with linalool (0.001–1000 nM) for 24 h. (**C**) Effect of MPP^+^ on cell viability of SH-SY5Y cells. SH-SY5Y cells were treated with different concentrations of MPP^+^ (100, 500, 1000 µM) for 24 h. (**D**) Effects of linalool on cell viability of SH-SY5Y cells. SH-SY5Y cells were treated with linalool (1 pM–1 µM) for 24 h. Cell viability was determined by MTT assay. Bars represent the mean ± SEM. Data represent the mean ± SEM from six independent experiments. # *p* < 0.05 vs. control group (vehicle only, 0.1% EtOH); * *p* < 0.05 vs. MPP^+^ only group.

Essential oils (EOs) have traditionally been employed for various medical purposes, including sedation, anxiety relief, antidepressant effects, and hypnotic properties [21]. Linalool (3,7-dimethyl-1,6-octadien-3-ol; Figure 1A), a natural monoterpene, is generally found in EOs of aromatic plants. Previous studies have demonstrated that linalool possesses antinociceptive, anticonvulsant, antioxidant, antidepressant, and anti-anxiety effects [22,23,24,25,26,27,28,29]. Drugs currently used in PD treatment include dopaminergic precursors, dopaminergic agonists, the inhibitors of catechol-O-methyl transferase, and monoamine oxidase B [30]. However, these anti-PD drugs have little clinical benefit in the treatment of nonmotor features, which are unfortunately often the most impact on the quality of life. Recent studies demonstrated that aromatherapy has therapeutic effects in neurodegenerative diseases, and the critical component responsible for the impact is linalool [31,32,33,34,35]. Linalool was indicated to produce an antidepressant-like effect through the interaction with the serotonergic pathway [36]. In addition, in Alzheimer-like mice, linalool reduced neurotoxicity and improved cognitive function by activating Nrf2/HO-1 and BDNF [27,37].

1-Methyl-4-phenyl-1,2,3,6-tetrahydropyridine (MPTP) is identified as a standard model of PD for its ability to induce numerous hallmarks of PD, including apoptosis, inflammation, energy failure, and oxidative stress in many mammals such as monkey and mice [9,38,39,40,41]. MPTP can pass into the BBB owing to its high lipophilicity and quickly transforms to its active metabolite 1-methyl-4-phenylpyridinium ion (MPP^+^) by monoamine oxidase B in glial cells, which then causes impairment to the nigrostriatal dopamine pathway and causes dopaminergic neuron damage [42,43]. Based on this evidence, we hypothesized that linalool might improve PD’s motor and nonmotor symptoms. Therefore, in this study, we evaluated the neuroprotective effects of linalool on dopaminergic SH-SY5Y cells, primary mesencephalic neurons, and cortical neurons against MPP^+^-induced apoptosis and oxidative stress. Additionally, we investigated the protective effects of linalool on MPTP-induced impairment of motor and nonmotor symptoms in a PD-like mouse model. 

## 2. Results

### 2.1. Linalool Increased the Cell Viability of MPP^+^-Treated SH-SY5Y Cells

First, we investigated the effect of linalool on the cell viability of SH-SY5Y cells using an MTT assay. Treatment with linalool (0.01–1 µM) for 24 h did not affect the cell viability of SH-SY5Y cells (Figure 1B). In contrast, when SH-SY5Y cells were treated with MPP^+^ (100, 500, 1000 µM) for 24 h, there was a significant concentration-dependent decrease in cell viability (Figure 1C). However, linalool treatment (0.1 nM–1 µM) attenuated MPP^+^-induced cytotoxicity (Figure 1D).

### 2.2. Linalool Decreased MPP^+^-Induced Apoptotic Cell Death and Neurite Retraction Accompanied by Increasing Neurotrophic Factors in SH-SY5Y Cells

**Figure 2:**
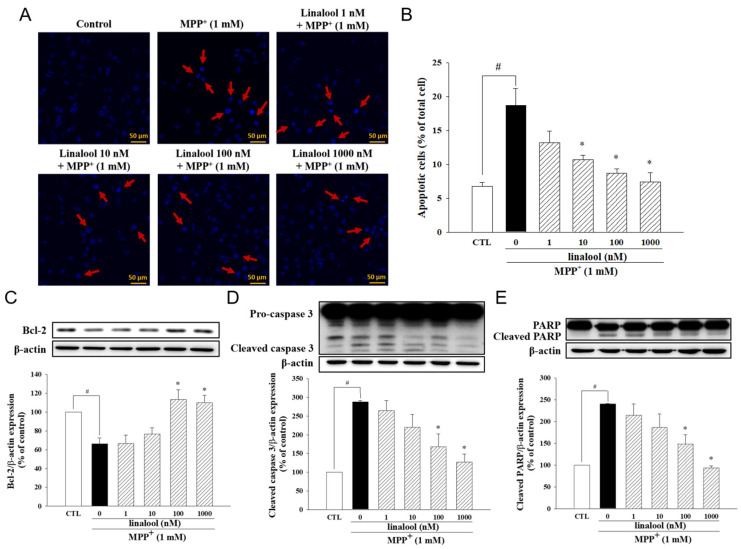
Linalool decreased MPP^+^-induced apoptotic death in SH-SY5Y cells. Cells were pretreated with linalool (1, 10, 100, and 1000 nM) for 1 h, then treated with MPP^+^ (1 mM) for 24 h. (**A**) Nuclear condensation (red arrow) was identified by Hoechst 33342 staining and observed by a fluorescent microscope. Scale bar = 50 µm. (**B**) The apoptosis degree was evaluated by counting the percentage of apoptotic cells (Hoechst 33342 staining). The protein expressions of (**C**) Bcl-2, (**D**) cleaved caspase-3, and (**E**) cleaved PARP were measured by Western blotting. Densitometry analyses are presented as the relative ratio of protein/β-actin and are represented as percentages of the control group. Data represent the mean ± SEM from six independent experiments. # *p* < 0.05 vs. control group (vehicle only, 0.1% EtOH); * *p* < 0.05 vs. MPP^+^ only group.

**Figure 3:**
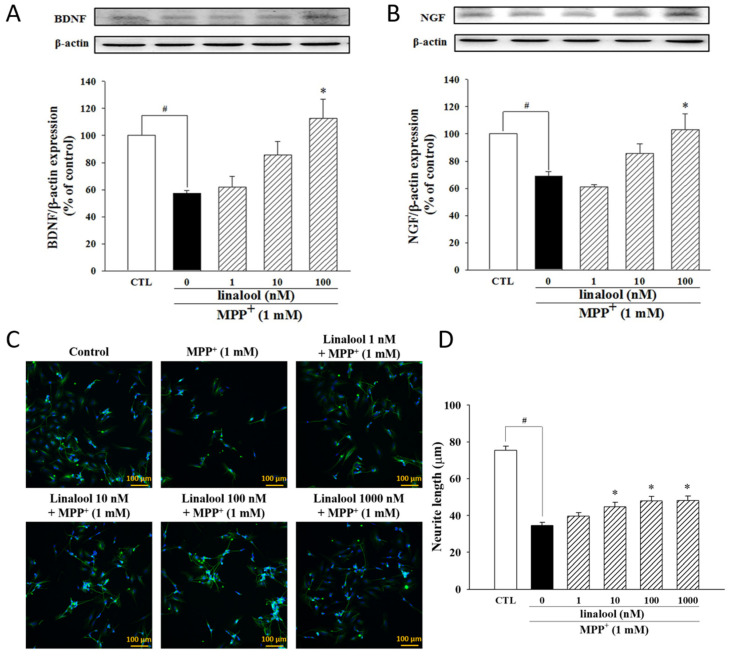
Linalool attenuated MPP^+^-induced reduction of neurite retraction in SH-SY5Y cells. Cells were pretreated with linalool (1–1000 nM) for 1 h and then treated with MPP^+^ (1 mM) for 24 h. To measure the neurite length, SH-SY5Y cells were differentiated in DMEM medium containing 3% FBS and 10 µM retinoic acid for five days before drug treatment. Densitometry analyses are presented as the relative ratio of (**A**) BDNF and (**B**) NGF protein/β-actin protein and are represented as percentages of the control or MPP^+^ group. (**C**) Neurite length and nuclear were confirmed by the fluorescent image of α-tubulin (green) and DAPI (blue) staining under a fluorescent microscope, respectively. Scale bar = 100 μm. (**D**) Neurite length was calculated by using Image J software version 1.54. Data were representing the mean ± SEM from six independent experiments. # *p* < 0.05 vs. control group (vehicle only, 0.1% EtOH); * *p* < 0.05 vs. MPP^+^ group.

The anti-apoptotic effects of linalool on MPP^+^-treated SH-SY5Y cells were examined by Hoechst 33342 staining. As shown in Figure 2A,B, MPP^+^-induced nuclear condensation and apoptotic death of SH-SY5Y cells, whose effects were inhibited by linalool (indicated by red arrows). Linalool also upregulated the expression of anti-apoptotic protein Bcl-2 (Figure 2C) and downregulated the expression of apoptotic protein cleaved caspase-3 (Figure 2D) and cleaved PARP (Figure 2E) in MPP^+^-treated SH-SY5Y cells. In addition, MPP^+^ significantly downregulated the neurotrophic protein BDNF (Figure 3A) and NGF (Figure 3B), which effects were reversed by linalool. We further investigated the protective effects of linalool on neurite length. As shown in Figure 3C,D, linalool attenuated MPP^+^-induced neurite retraction.

### 2.3. Linalool Enhanced Antioxidant Factors Nrf2/HO-1 and Inhibited NADPH Oxidase in MPP^+^-Treated SH-SY5Y Cells

**Figure 4:**
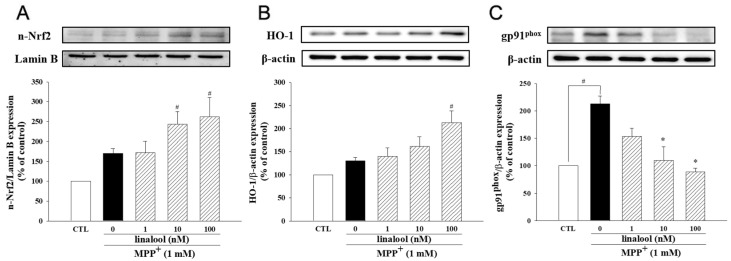
Linalool alleviated MPP^+^-induced the generation of oxidative stress in SH-SY5Y cells. Cells were pretreated with linalool (1, 10, and 100 nM) for 1 h and then treated with MPP^+^ (1 mM) for 24 h. Western blotting analyses are presented as the relative ratio of (**A**) nuclear-Nrf2/Lamin B, (**B**) HO-1/β-actin and (**C**) gp91^phox^/β-actin, and are represented as percentages of control or MPP^+^ group. Data represent the mean ± SEM from six independent experiments. # *p* < 0.05 vs. control group (vehicle only, 0.1% EtOH); * *p* < 0.05 vs. MPP^+^ group.

We further examine the antioxidative ability of linalool in MPP^+^-treated SH-SY5Y cells. In MPP^+^-treated cells, linalool increased the protein expression of nuclear Nrf2 and cytosolic HO-1, a critical factor meditating antioxidant character in neurons (Figure 4A,B). Moreover, we further examine the effects of linalool on the protein expression of gp91^phox^ (also known as NOX2), one of the catalytic subunits of nicotinamide adenine dinucleotide phosphate (NADPH) oxidase, which induces ROS production [44]. Results indicated that linalool downregulated the expression of gp91^phox^ in MPP^+^-treated SH-SY5Y cells (Figure 4C). These results suggested that linalool enhanced antioxidant defense in MPP^+^-treated SH-SY5Y cells. 

### 2.4. Linalool Enhanced the Expression of SirT1, Parkin, and TH in MPP^+^-Treated Primary Mesencephalic Neurons and the Expression of SirT1 and γ-Aminobutyric Acid (GABA) in MPP^+^-Treated Primary Cortical Neurons

**Figure 5:**
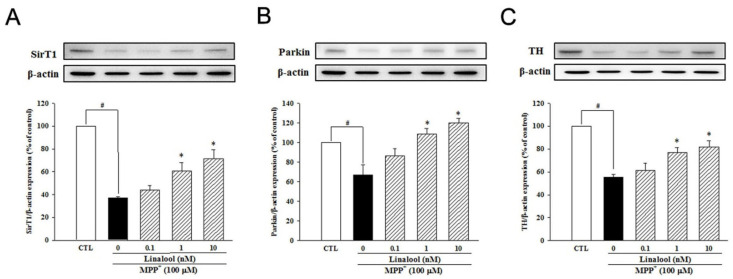
Effects of linalool on SirT1, parkin, and TH protein expression in MPP^+^-treated primary mesencephalic neurons. Primary mesencephalic neurons were pretreated with linalool (0.1, 1, and 10 nM) for 1 h and then treated with MPP^+^ (100 µM) for 24 hr. Linalool reversed the downregulation of (**A**) SirT1, (**B**) parkin, and (**C**) TH in MPP^+^-treated primary mesencephalic neuronal cultures. Densitometry analyses are presented as the relative ratio of protein/β-actin protein and are represented as percentages of the control group. Data represent the mean ± SEM from six independent experiments. # *p* < 0.05 vs. control group (vehicle only, 0.1% EtOH); * *p* < 0.05 vs. MPP^+^ group.

**Figure 6:**
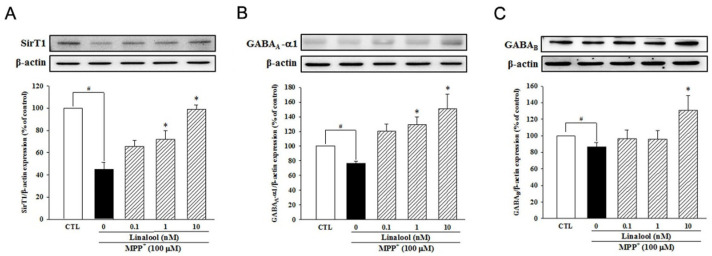
Linalool meliorated SirT1, GABA_A_-α1, and GABA_B_ protein expression in MPP^+^-treated primary cortical neurons. Primary cortical neurons were pretreated with linalool (0.1, 1, and 10 nM) for 1 h and then incubated with MPP^+^ (100 μM) for 24 h. Linalool attenuated MPP^+^-induced down-regulation of (**A**) SirT1, (**B**) GABA_A_-α1, and (**C**) GABA_B_ in primary cortical neurons. Western blotting analyses are presented as the relative ratio of protein/β-actin protein and are represented as percentages of the control group or MPP^+^ group. Data represent the mean ± SEM from six independent experiments. # *p* < 0.05 vs. control group (without any treatment); * *p* < 0.05 vs. MPP^+^ group.

Next, we examined the effects of linalool on the protein expression of sirtuin1 (SirT1)-related pathway in neuroprotection, depression, and anxiety. In primary mesencephalic neurons, MPP^+^ treatment decreased the protein expression of SirT1 (Figure 5A) and parkin (Figure 5B). However, pretreatment with linalool reversed the protein expression of SirT1 and parkin, which is involved in the neuroprotection against mitochondria-induced oxidative stress [45] in MPP^+^-treated primary mesencephalic neurons. In addition, linalool attenuated MPP^+^-induced reduction of TH protein expression (Figure 5C), a recognized marker of dopaminergic neurons. We further examined the expression of SirT1 and GABA in primary cortical neurons, which are involved in mood disorders like depression and anxiety. Our result indicated that MPP^+^ decreased the expression of SirT1 (Figure 6A), GABA_A-_α1 (Figure 6B), and GABA_B_ (Figure 6C) in the primary cortical neuron. However, linalool upregulated protein expression of SirT1, GABA_A_-α1, and GABA_B_ in MPP^+^-treated primary cortical neurons.

### 2.5. Linalool Protected Dopaminergic Neurons and Improved Motor Activity in MPTP-Induced Mouse Model of PD

**Figure 7:**
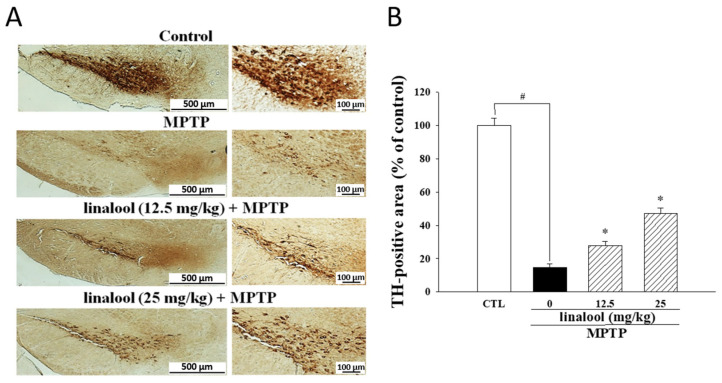
Linalool prevented MPTP-induced dopaminergic neuronal death in mice. (**A**) The immunohistochemical staining was performed to observe TH-positive neurons in the SNpc of MPTP-treated mice. Scale bar on the left panel of subfigure (**A**) = 500 µm. Scale bar on the right panel of subfigure (**A**) = 100 µm. (**B**) Quantitation of the intensity of TH staining. Data represent the mean ± SEM from six independent experiments. # *p* < 0.05 vs. control group (CTL, vehicle only); * *p* < 0.05 vs. MPTP group.

A dramatic decrease of dopaminergic neurons (TH-positive neurons) in the SNpc of MPTP-treated mice was observed (Figure 7A,B) compared to the control group. However, linalool reversed the reduction of TH-positive neurons in MPTP-treated mice (Figure 7A,B).

**Figure 8:**
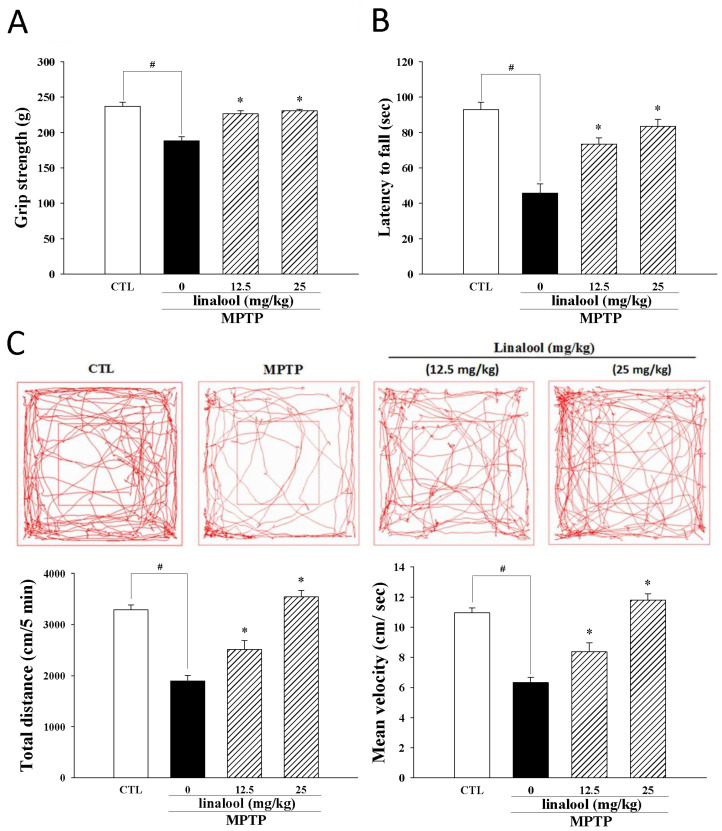
Linalool improved muscle strength and motor function in PD-like mice induced by MPTP. (**A**) The grip strength and (**B**) wire hang test were utilized to evaluate muscle strength. (**C**) The open-field test evaluated locomotor activity by measuring trace paths, total distance traveled, and the mean velocity. Data represent the mean ± SEM from six independent experiments. # *p* < 0.05 vs. control group (CTL, vehicle only); * *p* < 0.05 vs. MPTP group.

To evaluate the protective effect of linalool on motor neurological dysfunction in the PD model, the grip strength and wire hang test are utilized to examine muscle strength. Data from the study reported that PD-like mice have poor grip strength (Figure 8A) and shorter hanging times (Figure 8B). The muscle strength of PD-like mice was improved in the groups treated with 12.5 and 25 mg/kg of linalool. Moreover, the results of the open-field test indicated that the distance of total movement of PD-like mice was significantly reduced when compared to the control group. However, linalool increased the distance of total movement (Figure 8C), demonstrating that linalool improved locomotor activity in PD-like mice.

### 2.6. Linalool Improved Depressive- and Anxiety-like Behaviors in the MPTP-Induced Mouse Model of PD

**Figure 9:**
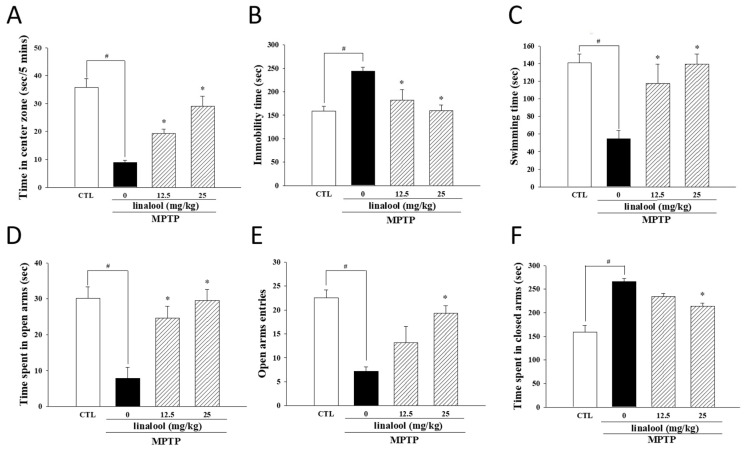
Linalool reversed MPTP-induced depressive- and anxiety-like behaviors in PD-like mice. (**A**) Time spent in the central area in the open-field test, (**B**) immobility time, and (**C**) swimming time in the forced swim test were used to measure depressive-like behavior. Elevated plus maze test was used to assess the anxiety-like behavior by recording (**D**) time spent in open arms, (**E**) the number of entries into open arms, and (**F**) time spent in closed arms. Data represent the mean ± SEM from six independent experiments. # *p* < 0.05 vs. control group (CTL, vehicle only); * *p* < 0.05 vs. MPTP group.

To assess the improved effect of linalool on nonmotor symptoms of PD, the open-field test, forced swim test, and the elevated plus maze test—three behavioral paradigms of depression—were performed. The results from the open-field test showed that time spent in the central area of PD-like mice was markedly decreased when compared to the control group. However, linalool improved the time spent in the central area (Figure 9A). Furthermore, MPTP increased the immobility time (passive behavior) in the force swimming test. However, the treatment of linalool (12.5 or 25 mg/kg) attenuated immobility time in MPTP-treated mice (Figure 9B). Moreover, time spent swimming or climbing (active behavior) was diminished in MPTP-treated mice, whereas those treated with linalool elongated the swimming time (Figure 9C). Anxiety and depressive disorders are often comorbid with PD [46]. Thus, an elevated plus maze test was assessed to measure anxiety symptoms in mice. The MPTP-induced anxiogenic effects, as shown by reduced time spent in the open arms (Figure 9D), decreased the number of entries into open arms (Figure 9E) and increased the time spent in closed arms (Figure 9F) in the elevated plus maze test. After applying linalool, the time spent in the open and the number of entries into open arms were markedly raised (Figure 9D,E), whereas the time spent in closed arms was decreased (Figure 9F). Taken together, these data provided evidence that linalool attenuated depressive- and anxiety-like behavior in PD-like mice induced by MPTP.

## 3. Discussion

Alzheimer’s disease (AD) and PD stand out as two highly common neurodegenerative disorders worldwide. Both conditions are linked to irregularities in the central nervous system and cellular damage, leading to diverse levels of cognitive and motor dysfunctions. Linalool is a naturally occurring terpene alcohol commonly found in various plants. Some studies suggest that linalool may have potential therapeutic properties, such as antioxidant and anti-inflammatory effects, which could be especially beneficial in neuroprotection. Previous studies indicated that administering linalool orally for three months reverses the histopathological hallmarks of AD and restores cognitive and emotional functions through an anti-inflammatory effect in aged triple transgenic AD mice model (3xTg-AD) [47]. The study by Yuan et al. in 2021 indicated that linalool acts against the neurotoxic effects of amyloid β (Aβ) in an AD fly model expressing human Aβ by suppressing free radical production and inflammatory response. In the unilateral Aβ_1–42_ lesion rat model of AD, linalool also reduced the initiation of oxidative stress and gliosis caused by Aβ_1–42_ treatment in the rat hippocampus [48]. Linalool also reverses the cognitive deficits through the alleviation of apoptosis and oxidative stress, dependent on the activation of Nrf2/HO-1 signaling in Aβ_1–40_-treated AD mice model [49]. Thus, linalool is anticipated to be utilized as a preventive or therapeutic agent for AD. In the present study, we reveal linalool possesses novel neuroprotective effects via regulating anti-apoptotic, antioxidative, and neurotrophic pathways. Linalool significantly increased the survival of dopaminergic neurons in SNpc and improved the impairments of skeletal muscle strength and motor/nonmotor symptoms in MPTP-induced PD-like mice.

MPP^+^ has been demonstrated to inhibit neurite outgrowth, decrease the number of neuron cells, and reduce the neurite length [50]. However, linalool attenuated the MPP^+^-induced neurite retraction in SH-SY5Y cells. Neurotrophic factors BDNF and NGF play important roles in regulating neuronal survival, differentiation, and functions [11,51]. In our result, linalool upregulated the protein expression of neurotrophic factors BDNF and NGF, which decreased with MPP^+^ in SH-SY5Y cells. Several studies indicated that MPP^+^-induced oxidative stress has been considered as one of the prominent detrimental effects that induce the toxicity of neurons in PD [52,53]. Meanwhile, the Nrf2/HO-1 pathway is a promising PD therapeutic target [54]. Our results indicated that linalool may regulate the effects of the Nrf2/HO-1 pathway in MPP^+^-treated SH-SY5Y cells. In addition, multiple evidences have suggested that NOX enzymes transport electrons across the plasma membrane, which enhances ROS production to induce the death of neuronal cells in the pathological processes of PD [55]. In the present study, our results illustrated that linalool significantly alleviated the upregulation of NOX2 expression caused by MPP^+^ in SH-SY5Y cells, implying the neuroprotective effects of linalool through the modulation of the oxidative stress system. A recent study has demonstrated the neuroprotective effect of SirT1 through the alleviation of oxidative stress generation in PD [56]. Moreover, parkin has been reported to be involved in the neuroprotective role of SirT1 in oxidative stress-induced mitochondrial dysfunction [45]. Cells from primary culture can more stably maintain the original shape, biological characteristics, and gene expression profiles of the cells. Therefore, primary mesencephalic neurons were used to further investigate the protective effect of linalool on MPP^+^-induced reduction of SirT1, parkin and TH. Our results suggested the neuroprotective effects of linalool through the up-regulation of SirT1 and parkin against the neurotoxicity induced by parkinsonian neurotoxin MPP^+^ in primary mesencephalic neurons. In addition, neurotrophic factors such as BDNF maintain neuronal plasticity and avoid oxidative stress-induced neuronal damage [10]. Results from this study demonstrated that linalool dose-dependently attenuated the MPP^+^-mediated neurotoxicity accompanied by upregulating antioxidant defense (Nrf2 and HO-1) and neurotrophic signaling (BDNF and NGF). These results supported that linalool possesses anti-oxidative and neurotrophic effects against MPP^+^-induced neurotoxicity in SH-SY5Y cells. Moreover, TH is the rate-limiting enzyme that catalyzes the hydroxylation of tyrosine to L-DOPA and is recognized as the marker of dopaminergic neurons. Linalool promotes TH expression in MPP^+^-treated primary mesencephalic neurons and PD-like mice, suggesting the protective potential of linalool on dopaminergic neurons.

The motor symptoms in PD become apparent when a significant portion of the dopamine neurons in the SNpc are lost. Additionally, the nonmotor symptoms, including neuropsychiatric, autonomic, sleep, and sensory disturbances, further affect the PD patient’s quality of life [57]. Neurotrophic factors have been established to play critical roles in the survival of neuronal cells in PD. BDNF and NGF were dramatically diminished in the SNpc of the human brain inflicted with PD. Therefore, the agonist of growth factors could be another option to mitigate PD pathology. Meanwhile, the reductions of plasma levels of BDNF and NGF have been shown in bipolar disorder, and manic and depressed patients [12,13,14]. Therefore, our results demonstrated that linalool rescued the BDNF and NGF expression in MPP^+^-induced neurotoxicity in SH-SY5Y cells and improved depressive- and anxiety-like behaviors in MPTP-induced PD. The nonmotor symptoms of PD usually respond poorly to dopaminergic treatment. Except for the loss of dopamine, the changes in other neurotransmitters like glutamate and GABA have participated in the pathogenesis of non-motor symptoms in PD [58]. Furthermore, pharmacological and genetic studies have demonstrated that the hypofunction of GABA-receptor results in the pathologies of schizophrenia, bipolar disorder, and major depressive disorder [59,60,61,62]. The change in GABA is also observed in the pathogenesis of nonmotor symptoms in PD [63]. Moreover, the SirT1 pathway is involved in the neuroprotection, depression, and anxiety in models of PD [56,64,65]. The activation of SirT1 plays a role in modulating the secretion of GABA [66,67]. Previous reports have shown that linalool has soothing effects to counteract anxiety [22]. Our results indicated that linalool enhanced SirT1 expression in MPP^+^-treated primary cortical neurons and protected against MPP^+^-induced cytotoxicity in SH-SY5Y cells and primary rat embryonic neuronal cells. Moreover, linalool attenuated MPTP-induced motor impairment in C57BL/6 mice, such as exercise ability, grip strength and muscle endurance, as well as nonmotor symptoms, such as depression and anxiety symptoms. These findings reveals the potential benefits in the complementary therapy for PD. 

## 4. Materials and Methods

### 4.1. Chemicals and Antibodies

Linalool, (3-(4,5-dimethylthiazol-2-yl)-2,5-diphenyl-tetrazolium bromide) (MTT), MPP^+^, MPTP, Hoechst 33342 and mouse antibody against β-actin were obtained from Sigma-Aldrich (St. Louis, MO, USA). DMEM, FBS, and Alexa Fluor 488 goat anti-rabbit IgG were purchased from Invitrogen (Carlsbad, CA, USA). Mouse antibodies against Bcl-2, goat antibody against HO-1 and all horseradish peroxidase-conjugated secondary antibodies were acquired from Santa Cruz Biotechnology (Santa Cruz, CA, USA). Mouse antibody against caspase-3 and rabbit antibody against PARP were obtained from Cell Signaling (Danvers, MA, USA). Mouse antibody against gp91^phox^ was purchased from BD Bioscience (San Jose, CA, USA). Rabbit antibody against α-tubulin, rabbit antibody against TH, enhanced chemiluminescence reagent, and polyvinylidene difluoride (PVDF) membrane were procured from Millipore (Bedford, MA, USA). All materials for SDS-PAGE were acquired from Bio-Rad (Hercules, CA, USA).

### 4.2. Cell Cultures

Human SH-SY5Y dopaminergic cells were procured from the American Type Culture Collection (ATCC). SH-SY5Y cells were incubated in DMEM growth medium containing 10% FBS, 100 U/mL penicillin, and 100 μg/mL streptomycin in a humidified incubator with 5% CO_2_ at 37 °C. To assess the protective impact of linalool against MPP^+^-induced damage, SH-SY5Y cells were pretreated with vehicle (0.1% EtOH) or linalool (1, 10, 100, 1000 nM) for 1 h, followed by exposure to MPP^+^ (1 mM) for 24 h. Primary cortical and mesencephalic neurons were prepared from cortices and ventral mesencephalon dissected from fifteen-day-old embryos (E15) of SD rats, respectively [68]. After 6 days in vitro, cultured cells were treated with various concentrations of linalool in the experiment. To evaluate the protective effects of linalool against MPP^+^-induced damage, primary cortical and mesencephalic neurons were pretreated with vehicle (0.1% EtOH) or linalool at concentrations of 0.1, 1 and 10 nM for 1 h. Subsequently, the cells were exposed to MPP^+^ (100 μM) for 24 h.

### 4.3. Cell Viability Assay

Cell viability was determined by the MTT assay. Cells were treated with 0.5 mg/mL MTT for 3 h in 37 °C, and then the formazan crystals in the cells were solved with 100 μL of DMSO (Phillipsburg, NJ, USA). The absorbance was read at 540 nm and 630 nm by using a microplate reader.

### 4.4. Apoptotic Nuclear Condensation

Apoptotic nuclear condensation as detected by Hoechst 33342 staining. The SH-SY5Y cells were seeded into 6-well plates and then treated as described above. After treatments, Hoechst 33342 (10 μM) was added to incubate for 10 min. Images were obtained using a fluorescence microscope (Nikon, Tokyo, Japan). The apoptosis degree was evaluated by counting the percentage of apoptotic cells.

### 4.5. Measurement of Neurite Length

To measure the neurite length, SH-SY5Y cells were differentiated in a DMEM medium containing 3% FBS and retinoic acid (10 µM) for 5 days before drug treatment. α-tubulin staining was assessed to measure neurite outgrowth by measuring the length of neurite for all identified positive neurite-bearing cells and calculating the mean of neurite length per cell using ImageJ software version 1.54 (National Institutes of Health, Bethesda, MD, USA). Briefly, cells were fixed with 4% paraformaldehyde for 30 min and permeabilized with 0.2% Triton X-100 (USB Corporation, Cleveland, OH, USA) in PBS. Cells were then incubated with blocking buffer (2% BSA in PBS) for 1 h, and with rabbit anti-α-tubulin overnight at 4 °C. Alexa Fluor 488 goat anti-rabbit IgG was used as the secondary antibody. Images were collected under a fluorescence microscope (Nikon, Japan). Neurites were defined as processes with a length equal to or exceeding two cell body diameters. The analysis of neurite outgrowth involved measuring the length of neurites for all positively identified neurite-bearing cells. Subsequently, the average neurite length per cell was calculated [69,70].

### 4.6. Western Blot Analysis

After the indicated treatment, cells were collected and lysed to determine the protein expression. Cells were lysed in T-PER tissue protein extraction buffer to obtain total cell extract or NE-PER™ nuclear and cytoplasmic extraction reagents to isolate nuclear fraction. Collected protein was used for SDS-polyacrylamide gel electrophoresis, and protein concentration was determined using the Bio-Rad protein assay kit (Hercules, CA, USA). The membranes were incubated, each with indicated primary antibodies by different dilutions overnight at 4 °C. Membranes were then washed with TBST and incubated with secondary antibodies (diluted 1:10,000 in 5% nonfat milk), shaking at room temperature for 1 h. Protein expression was detected with chemiluminescence using an Amersham ECL detection kit (Cytiva, Marlborough, MA, USA), and the signal was captured using the TopBio Multigel-21 chemiluminescent imaging system (Topbio, Taiwan) and quantified with the ImageJ software version 1.54 (National Institutes of Health, Bethesda, MD, USA).

### 4.7. Animal Groupings and Drug Administration

The Animal Care and Use Committee at the Kaohsiung Medical University approved the animal use and methods (IACUC-104153; date of approval: 21 January 2016). Male C57BL/6 mice (7–8 weeks old, 20–25 g) were purchased from the National Laboratory Animal Breeding and Research Center (Taipei, Taiwan). For oral administration to mice, linalool was dissolved in normal saline containing 0.2% Tween 80. Mice were randomly separated into four groups (six mice per group) as follows: control (vehicle); MPTP (MPTP + vehicle); MPTP + linalool (12.5 mg/kg/day); MPTP + linalool (25 mg/kg/day). Mice were pre-treated with vehicle (5 mL/kg) or linalool (12.5 mg/kg/5 mL or 25 mg/kg/5 mL, once daily for 7 days, and then MPTP was injected on the 8th day. MPTP-induced subacute PD mice model was constructed in the present study. Mice were administered 20 mg/kg MPTP via intraperitoneal injection four times at 2 h intervals within a day.

### 4.8. Grip Strength Test

A grip strength meter (Bio-GS3, BioSeb, Vitrolles, France) was used to evaluate the muscle strength of the whole limb muscle force; the grip strength test of the mice was conducted following the methodology described in our previous reports [71,72,73] and research reports from other labs [74,75]. Before the experiment, mice were acclimated to the new environment for 2 h. For each trial, mice were placed on the grid of the device and pulled parallel to the ground at a constant speed until loss of grip and the maximum force generated just before the mouse lost its grasp was recorded. This process was replicated three times for three cycles, and each trial was followed by a 5-s resting period of the mouse on the benchtop. The mice were allowed to rest for 10 min between every cycle. The average maximum limb muscle strength values in grams (g) were used in the evaluation.

### 4.9. Wire Hang Test

The wire hang test assessed neuromuscular strength [76]. Before the experiment, mice were acclimated to the new environment for 2 h. After that, mice were placed on a horizontally stretched line until they grasped the line with four legs. The wire was installed 30 cm above the ground and is cushioned underneath to prevent injury caused by falling. To assess the neuromuscular strength of the whole limb muscle force, we conducted nine consecutive experiments for each mouse. The mice were allowed to rest between tests. The average latency to fall values in seconds (s) was used in the evaluation.

### 4.10. Open-Field Test

The open-field test was used to measure locomotor activity and anxiety-like behavior [40,72,77]. The apparatus is composed of a chamber (50 × 50 cm in diameter, 25 cm in height). Before the experiment, mice were acclimated to the new environment for 2 h and were placed in a chamber for 5 min, followed by automatic measurement by a DigiScan analyzer (ViewPoint behavior technology, Civrieux, France), which transmitted the activity data to a computer. The activity data presented was the total distance (cm) of movement, mean velocity (cm/s) and the time in the center zone within the 5-min recording period.

### 4.11. Elevated plus Maze Test

The elevated plus maze test was used to assess anxiety-like behavior [77]. The apparatus is composed of a central square (5 × 5 cm), two open arms (30 × 5 cm), and two closed arms with walls (30 × 5 × 15 cm). The activity data presented was time spent in open arms, number of entries into open arms and time spent in closed arms within the 5-min recording period.

### 4.12. Forced Swimming Test

The forced swimming test was used to assess depression-like behavior [36]. The apparatus is composed of a plastic cylinder (12 cm in diameter, 25 cm in height). The cylinder was filled with water (24 ± 1 °C) up to 15 cm height. Before the experiment, mice were acclimated to the new environment for 2 h. After that, mice were placed in the cylinder for 6 min (1 min to adapt to the environment and 5 min to register immobility time), followed by automatic measurement by a DigiScan analyzer (ViewPoint behavior technology, Civrieux, France), transmitting the activity data to a computer. The activity data presented was immobility time within the 5-min recording period.

### 4.13. Immunohistochemistry

In immunohistochemistry, brains were first perfusion-fixed with 4% paraformaldehyde and then post-fixed overnight after dissection. The brain tissues were dehydrated and embedded in paraffin wax, and 5-µm-thick consecutive coronal sections were collected. The sections were then incubated with 0.3% H_2_O_2_ for 15 min to exhaust endogenous peroxidase activity. Subsequently, the slides were incubated with a blocking buffer containing 3% bovine serum albumin (BSA) for 30 min. The tissue sections were incubated overnight with the primary antibody TH (1:100) at 4 °C. After washing with PBS, the sections were incubated with an antirabbit HRP-conjugated secondary antibody (1:500) for 30 min at room temperature. The sections were then developed with diaminobenzidine (DAB). TH-positive neurons in the SNpc were captured under an optical microscope (ECLIPSE 80i, Nikon, Japan) at a magnification of 200×. The ability of neurons in the SNpc to synthesize dopamine was evaluated by quantifying the TH-positive neuronal cells in the SNpc.

### 4.14. Statistical Analysis

Statistical analysis was performed using InStat version 3.0 (GraphPad Software, San Diego, CA, USA). Data were expressed as means ± SEM and statistical significance was analyzed using one-way ANOVA followed by Dunnett’s test for all pair comparisons. Significance was set at a *p*-value of less than 0.05.

## 5. Conclusions

Our results demonstrated that linalool attenuated MPP^+^-induced apoptotic death, neurite damage, and oxidative stress. Meanwhile, linalool improved not only the motor function of PD-like mice but also the nonmotor function. These findings provide novel evidence-based insight, supporting the potential use of linalool or linalool-containing EOs as a complementary therapy for PD.

## Data Availability

The data presented in this study are available on request from the corresponding author.
